# Leveraging topoisomerase II-mediated DNA damage: repurposing etoposide as a lead compound for apicomplexan parasite control

**DOI:** 10.3389/fvets.2025.1689833

**Published:** 2025-11-19

**Authors:** Yongchang Li, Zeyun Cui, Jianlong Li, Yang Zhang, Iqra Zafar, Mohamed Abdo Rizk, Hang Li, Shimaa Abd El-Salam El-Sayed, Naoaki Yokoyama, Qingyong Guo, Xuenan Xuan, Bayin Chahan

**Affiliations:** 1 Parasitology Laboratory, Veterinary College, Xinjiang Agricultural University, Urumqi, Xinjiang, China; 2 National Research Center for Protozoan Diseases, Obihiro University of Agriculture and Veterinary Medicine, Obihiro, Hokkaido, Japan; 3 Laboratory of Sustainable Animal Environment, Graduate School of Agriculture Science, Tohoku University, Sendai, Japan; 4 Department of Internal Medicine and Infectious Diseases, Faculty of Veterinary Medicine, Mansoura University, Mansoura, Egypt; 5 Department of Biochemistry and Chemistry of Nutrition, Faculty of Veterinary Medicine, Mansoura University, Mansoura, Egypt; 6 Research Center for Asian Infectious Diseases, The Institute of Medical Science, The University of Tokyo, Tokyo, Japan

**Keywords:** bovine babesiosis, equine piroplasmosis, etoposide, *in vitro*, *in vivo*, inhibitory effects

## Abstract

*Babesia* and *Theileria* are microscopic parasites that infect livestock, leading to substantial economic losses. Current treatments are often limited by challenges such as drug resistance and incomplete parasite eradication. This study investigates potential of Etoposide (EP), a well-known anticancer drug that disrupts DNA Topoisomerase II, as a treatment for these parasitic infections. The research focused on EP’s ability to inhibit *Babesia bovis*, *Babesia caballi*, and *Theileria equi*, evaluating its impact on parasite viability, structural changes, and protective role for host red blood cells. Parasites were exposed to various concentrations of EP (ranging from 0.50 × to 4 × IC_50_), and their viability and morphology were assessed through bioassays and Giemsa-stained slides analysis. *In vivo* experiments were conducted using a mouse model infected with *Babesia microti*, to examine changes in parasite burden, red blood cell counts, and fluorescence-based signals. The results demonstrated that EP inhibited parasite growth in a dose-dependent manner, with IC_50_ values of 11.23 ± 2.82 μM for *B. bovis*, 0.037 ± 0.039 μM for *B. caballi*, and 0.68 ± 0.39 μM for *T. equi*. Importantly, parasites treated with EP did not recover when returned to untreated culture conditions. Morphological changes included distinct spots in *B. bovis* and *B. caballi*, along with abnormal structures in *T. equi*. These findings suggest that EP has potential as a complementary therapy, enhancing the effectiveness of existing treatments for *Babesia* and *Theileria* infections. Further research is warranted to refine its application and investigate its role in combination therapy strategies.

## 1 Introduction

*Babesia* spp. are globally distributed, tick-borne protozoan parasites that cause significant diseases in livestock, such as bovine babesiosis in cattle and equine piroplasmosis in horses, resulting in substantial economic losses (1). In cattle, *Babesia bovis* and *Babesia bigemina* are the primary causative agents leading to symptoms that include high fever, hemolytic anemia, hemoglobinuria, and, in severe cases, death (2). In horses, equine piroplasmosis is caused by *Babesia caballi* and *Theileria equi*, which induce similar clinical signs (3). Although significant progress has been made in developing drugs targeting *Babesia*, further research and innovation remain essential (4).

DNA topoisomerase II (TopoII) is indispensable for chromosome dynamics in rapidly dividing protozoan parasites, resolving topological stress through transient double-stranded DNA break formation and religation (5). Critically, structural and functional distinctions exist between parasitic TopoII isoforms and human topoisomerases (hTop) (6), enabling selective targeting for antiprotozoal drug development. This therapeutic potential is evidenced by: (i) Stage-specific expression of Plasmodium PfTopoVIB and PfSpo11 during late schizogony, coinciding with mitochondrial genome segregation (7); (ii) Dual-stage antiplasmodial activity of (ii) Dual-stage antiplasmodial activity of *Aloe marlothii* constituents, with aloesaponarin I and aloesaponol IV computationally identified as *β*-hemoglobin and TopoII inhibitors, respectively (8); (iii) Lethal DNA damage in parasites induced by etoposide (EP)-mediated stabilization of TopoII-DNA cleavage complexes (9).

Recent studies have expanded the scope of drug research to include anti-inflammatory, antibacterial, anti-tumor, and immunomodulatory effects. Novel hybrid compounds, such as 7-chloro-4-aminoquinoline-benzimidazole (10) and monoanionic gold bis (dithiolene) complexes (11) demonstrate this paradigm shift. However, application to protozoal diseases beyond malaria, toxoplasmosis, and trypanosomiasis—prioritized for their zoonotic significance application to protozoal diseases beyond malaria, toxoplasmosis, and trypanosomiasis—prioritized for their zoonotic significance (12). Effective babesiosis control is clinically imperative (13) particularly with emerging pathogens like *Babesia duncani* (14) alongside established agents *B. microti* and *B. divergens*. This study provides mechanistic evidence supporting antitumor drug repurposing against Babesia species. Collectively, these insights advance understanding of blood-protozoan pathogenesis and potential hematological implications.

## 2 Materials and methods

*Babesia bovis* (Texas strain), *B. caballi* (USDA strain), and *T. equi* (USDA strain) were cultured using purified bovine and horse red blood cells (RBCs) in GIT and RPMI 1640 media. Cultures were maintained in 24-well plates in a 37 °C in an incubator with a gas mixture comprising 5% CO_2_ and 5% O_2_. The culture medium was refreshed every 24 h (2). Concurrently, *B. microti* (Munich strain) was administered to 7-week-old female BALB/c mice (CLEA Japan Inc., Tokyo, Japan). The SYBR Green I (SGI) nucleic acid stain (Lonza, USA; 10,000 ×) and lysis buffer were prepared following the procedure described by Li et al. (2), solutions were stored at −30 °C and 4 °C, respectively. Stock solutions of diminazene aceturate (DA, Novartis, Japan), Etoposide (EP, Sigma-Aldrich, Japan) were prepared at a concentration of 100 mM and stored at −30 °C until use. Cell toxicity was evaluated using the Cell Counting Kit-8 (CCK-8, Japan). IC_50_ values for *B. bovis*, *B. caballi*, and *T. equi* were calculated for concentrations ranging from 0.10 to 250 μM using non-linear regression analysis (curve fit) in GraphPad Prism 7 (GraphPad Software Inc., USA). Non-infected RBCs and 0.5% dimethyl sulfoxide (DMSO) were used as blank and negative controls, respectively. Each compound concentration was tested in triplicate, and the experiment was repeated three times.

The viability of *B. bovis* treated with drugs was assessed using the protocol (2). In brief, 10 μL of 1% infected RBCs (iRBCs) and 90 μL of medium containing varying drug concentrations were dispensed into a 96-well plate. The plate was incubated under the previously established conditions, and the medium was replaced every 24 h for 4 days. Fresh medium containing the respective concentrations of EP (0.50×, 1×, 2×, and 4 × the IC₅₀) was added during each medium change. On the fifth day, 3 μL of RBCs from the treated wells were mixed with 7 μL of fresh RBCs in a new 96-well plate without the drug, and the medium was refreshed daily for the next 5 days. Thin blood smears were prepared and stained with Giemsa (GBS) on the fifth day. Each experiment was performed in triplicate and repeated three times. To evaluate cell viability in response to drug treatment, the Cell Counting Kit-8 (CCK-8, Japan) assay was utilized. In summary, Madin-Darby Bovine Kidney (MDBK) cells were seeded at a density of 5 × 10^4^ cells/mL in 96-well plates, with 100 μL of cell suspension per well, and incubated for 24 h. Next, 100 μL of drug solutions at various concentrations (5–1,000 μM) were added in triplicate to the wells. After a 24-h incubation period, 10 μL of CCK-8 reagent was added to each well followed by an additional 4-h incubation. Absorbance at 450 nm was then measured using an MTP-500 microplate reader (Corona Electric, Japan). Wells containing only culture medium served as blanks, while wells with cells in medium with 0.5% DMSO were used as controls (2).

The therapeutic efficacy of selected compounds was assessed using a mouse model infected with *B. microti*, following the protocol outlined by Rizk et al. (15). Thirty-five 7-week-old female BALB/c mice (From CLEA Japan Inc., Tokyo, Japan) were randomly divided into four groups. The first group received EP treatment at a dose of 1 mg/kg, while the second group was administered the standard drug DA at 25 mg/kg. The third and fourth groups were positive (Infected and untreated) and negative (Uninfected and untreated) controls, respectively. Treatments were delivered via intraperitoneal injection (IP) for five consecutive days (From days 4–8) after parasitemia levels reached 1%. Following previously study (2), a *B. microti*-positive mouse model was established by retrieving *B. microti* from the cell bank and I njecting into a mouse. Parasitemia was determined every other day by examining GBS under a 100 × oil immersion Eclipse E200 microscope (Nikon, Tokyo, Japan). The percentage of parasitized erythrocytes was calculated by counting 10^3^ erythrocytes. When parasitemia reached 30%, blood was collected, diluted in 1 × PBS to 2 × 10^7^ infected red blood cells (iRBCs)/mL, and 0.5 mL of this suspension was injected intraperitoneally into all groups, except the negative control. The final concentration of injected iRBCs was 1 × 10^7^/mL. Parasitemia was monitored bi-daily for 32 days, or until parasitemia was cleared using GBS as described earlier. Tail blood (2.5 μL) was collected every 2 days and analyzed in 96-well plates using RPMI 1640 medium, lysis buffer, and 2 × SGI stain, followed by fluorescence measurement after 1 h of incubation. Hematological analyses were performed every 4 days using a hemocytometer (Celltac *α* MEK-6450, NihonKohden, Japan). All experiments were carried out in duplicate.

The IC_50_ values for EP and DA were calculated using non-linear regression analysis with GraphPad Prism 6.0 (GraphPad Software Inc., USA). The half-maximal inhibitory concentration (IC₅₀) values for EP and DA were determined by non-linear regression analysis using GraphPad Prism 6.0 (GraphPad Software Inc., USA). Specifically, a four-parameter logistic model (4PL) was fitted to the dose–response data obtained from at least three independent experiments, each performed in technical triplicate. The model provides the IC₅₀ as the inflection point of the sigmoidal curve. All reported IC₅₀ values are presented as the mean ± standard error of the mean (SEM) derived from these independent replicates. This analysis included independent Student’s t-tests and one-way ANOVA. A *p*-value of less than 0.05 was considered statistically significant, with values below 0.01 indicated a highly significant difference. The study protocol received approval from the Committee on the Ethics of Animal Experiments at Obihiro University of Agriculture and Veterinary Medicine, Japan (Permit numbers: animal experiment, 230,244; DNA experiment, AP0001299621; Pathogen, AP0001299622; Ethical approval numbers, 22–23 and 23–17).

## 3 Results

**Figure 1:**
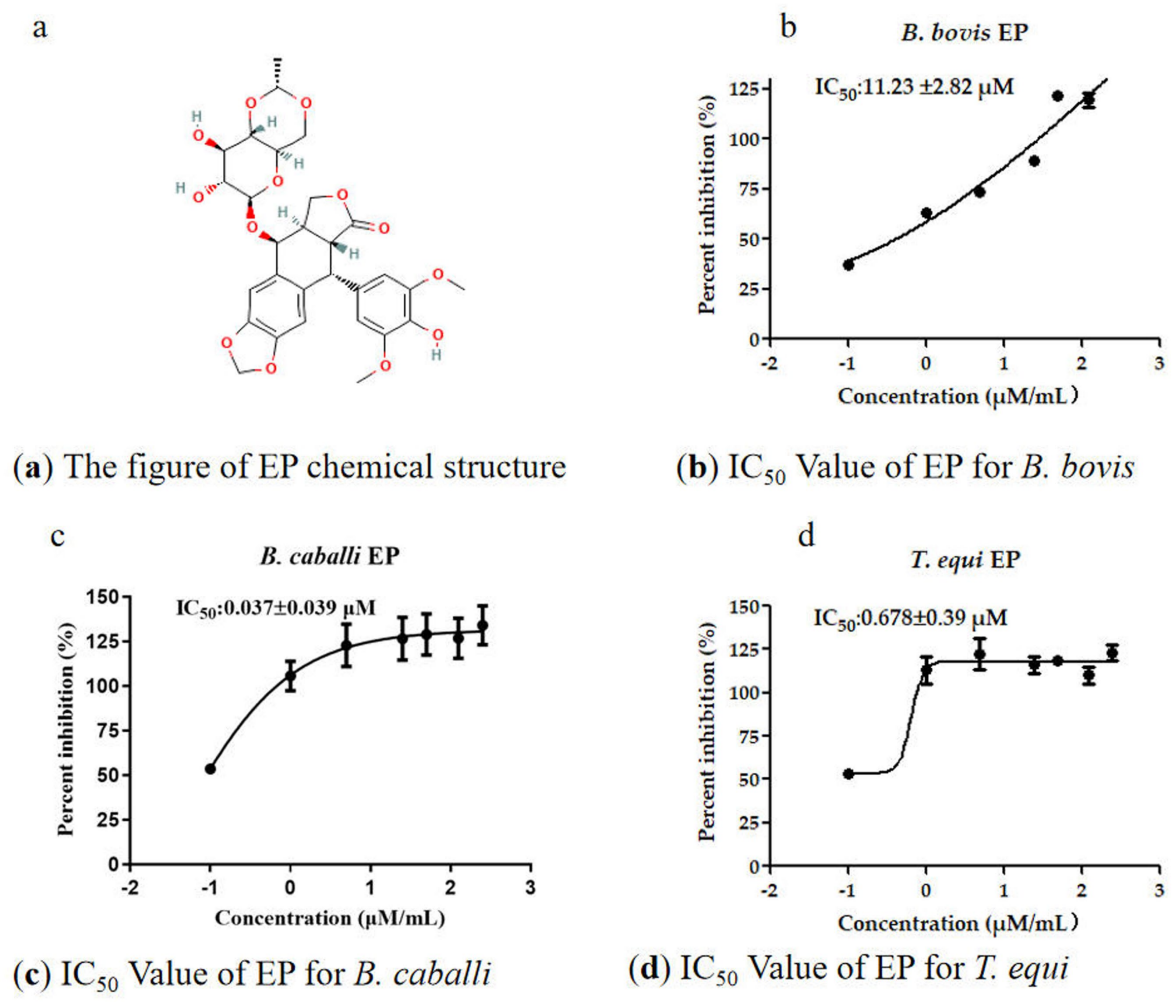
The information of EP and IC50 for three parasites in *in vitro*.

The EP demonstrated potent inhibitory effects on *B. bovis*, *B. caballi* and *T. equi* and the IC_50_ values for EP against these parasites were 11.23 ± 2.82 μM, 0.037 ± 0.039 μM, and 0.68 ± 0.39 μM, respectively (Figures 1a–d). To further validate the effectiveness of the identified EP as inhibitor of *B. bovis*, *B. caballi*, and *T. equi*, both viability assays and morphological assessments of treated cultures were performed. Following treatment with varying concentrations of EP (IC_50_ ranging from 0.50 × to 4×), no recovery of *B. bovis*, *B. caballi*, or *T. equi* was observed when the cultures were returned to standard medium conditions. GBS were used to examine parasitemia at 24 h, 72 h, and 5 days. All quantitative data are presented as the mean ± standard error of the mean (SEM) from at least three independent experiments, each performed in technical triplicate. Statistical analyses were conducted using one-way ANOVA followed by Tukey’s multiple-comparison test or Student’s *t*-test where appropriate. Statistical significance was annotated directly on the graphs as *p* < 0.05, *p <* 0.01, and *p* < 0.001. Error bars in all figures represent the SEM. Where no bars are visible, the errors are smaller than the symbols. All analyses were performed using GraphPad Prism 7.0 (GraphPad Software Inc., USA).

**Figure 2:**
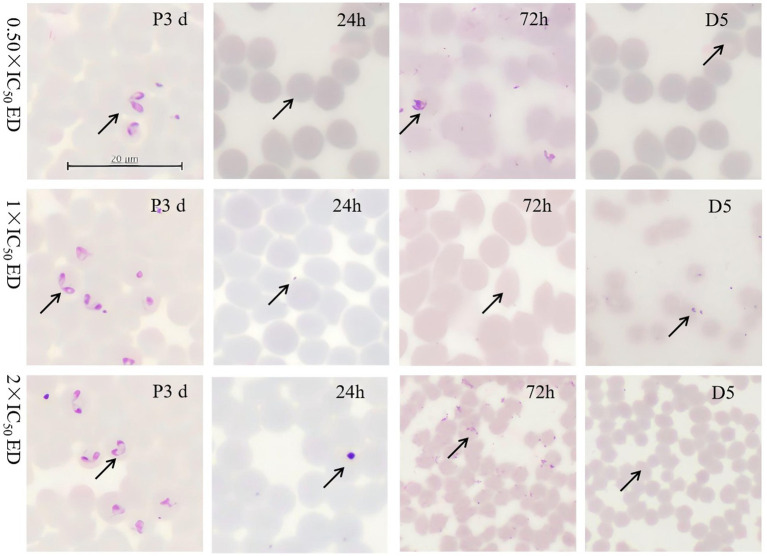
Morphological changes observed in 0.50×- 2 × EP treated *B. bovis*.

Morphological analysis revealed small spots within RBCs in *B. bovis* and *B. caballi* treated with 0.50 × IC_50_ of EP on day 5, compared to the positive control (P3d). In *T. equi*, suspected parasite bodies appeared at 72 h and on day 5 following treatment with 0.50 × and 2 × IC_50_. Notably, no regrowth of *B. bovis*, *B. caballi*, or *T. equi* was observed at EP concentrations of 11.23, 22.46, and 44.92 μM; 0.074, 0.148, and 0.296 μM; and 1.36, 2.72, and 5.44 μM, respectively (Figure 2, Supplementary Figures S1, S2).

Treatment with EP *in vitro* showed no cytotoxic effects on MDBK cells at concentrations up to 500 μM. Notably, EP, identified as a potent natural compound, exhibited a low IC_50_ value for MDBK cells while demonstrating a very high selectivity index (SI). The cytotoxicity assay results were used to calculate the selectivity index (SI = IC₅₀ for MDBK cells ÷ IC₅₀ for parasites), providing a comparative measure of safety. The SI values for EP were 44.5 for *B. bovis*, 13,514 for *B. caballi*, and 735 for *T. equi*, indicating a favorable safety margin across all tested species. For comparison, the standard anti-babesial drug diminazene aceturate (DA) exhibits reported SI values in the range of 250–500 under similar assay conditions (2). Thus, while EP’s antiparasitic potency is modest relative to DA, its markedly higher SI—especially against *B. caballi* and *T. equi*—suggests low mammalian cytotoxicity and potential utility as a lead compound for safer combination therapy development.

**Figure 3:**
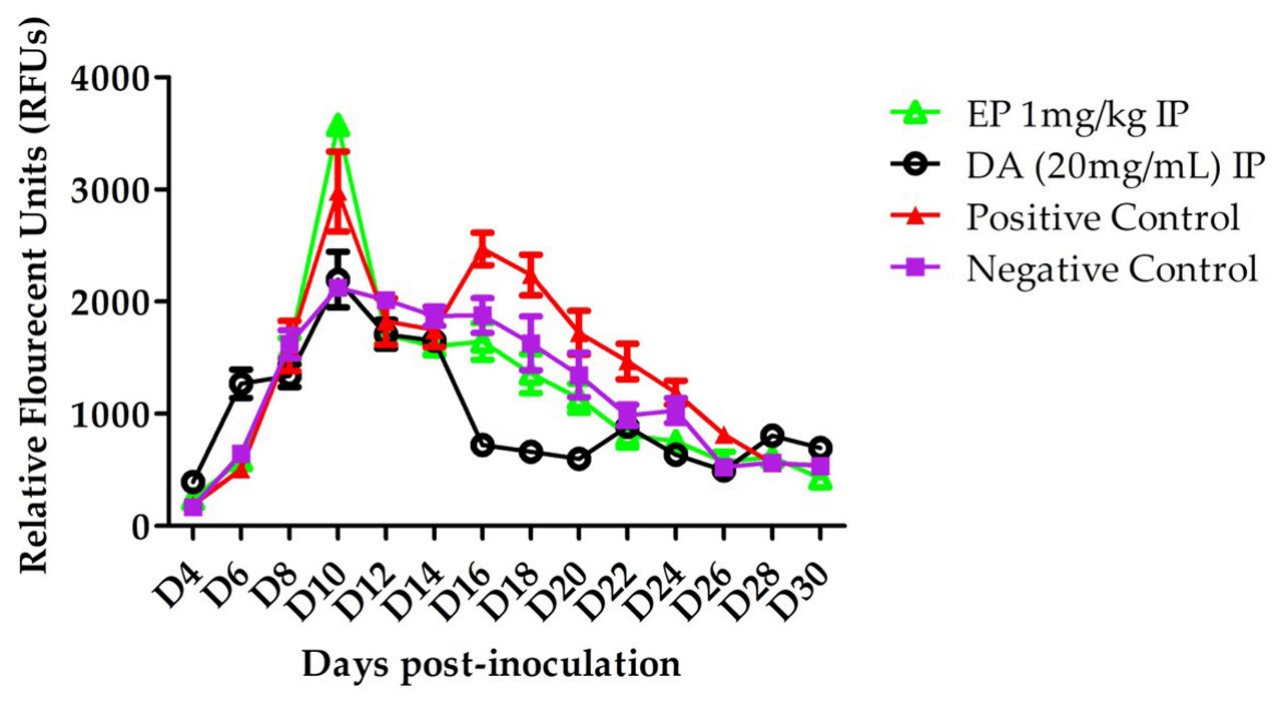
The White blood cells (WBCs), RBCs, HCT and HGB of EP in *B. microti in vivo*.

The *in vivo* efficacy of EP against *B. microti* was evaluated. In the EP treated group, a slower increase in parasitemia was observed following intraperitoneal (IP) injections on days 14, 16, and 18. However, statistical analysis revealed no significant difference compared to the control group (*p* > 0.05). On day 16, relative fluorescence units (RFUs) values for the treatedgroup were recorded at 1,800 for 1 mg/kg EP and 960 for 20 mg/kg DA, whereas the control group exhibited a peak RFU of 2,500 (Figure 3).

**Figure 4:**
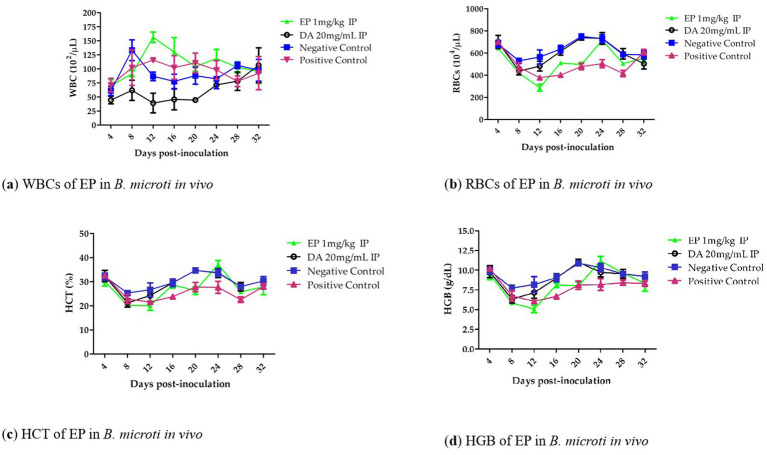
The White blood cells (WBCs), RBCs, HCT and HGB of EP in *B. microti in vivo*.

Notably, hematological parameters including RBC count (Figure 4a), hemoglobin (HGB) concentration (Figure 4b), hematocrit (HCT) percentage (Figure 4c), and HGB count (Figure 4d), showed an increasing trend from days 12 to 20 in the EP-treated groups when compared to the untreated infected group. However, no statistically significant reduction (*p* > 0.05) in RBC count, HGB concentration, or HCT percentage was observed in the EP-treated groups relative to the DA-treated group.

## 4 Discussion

Tick-borne diseases (TBDs) in animals are increasingly prevalent in subtropical and tropical regions; however, research focusing on Asian countries remains limited (16). Babesiosis, one of the TBDs, is primarily transmitted by Ixodid ticks (17, 18). As a region adjacent to Xinjiang, Pakistan continues to report cases of bovine babesiosis and theileriosis. For instance, Kebzai et al. (19) detected *Theileria annulata* in cattle blood samples from Loralai and Zhob districts, with infection rates of 12.75 and 12.25% via PCR. Notably, the prevalence among young cattle reached 85.82% (19). Additionally, *T. annulata* has been found to infect camels in Pakistan, showing an overall infection rate of 13.5% (20).

In recent years, several new therapeutic approaches have been developed for bovine theileriosis and babesiosis, including Juglone, Buparvaquone (21), imidocarb dipropionate, and the MCD protocol (metronidazole, clindamycin, and doxycycline) (19, 22). Following treatments such as Juglone and Buparvaquone, a decrease in total leukocyte and lymphocyte counts was observed in recovering cattle (21). In *T. equi* infections, affected horses exhibited reduced levels of total and free testosterone, as well as tri-iodothyronine (T3), alongside elevated thyroxine (T4) and cortisol levels compared to healthy animals (23). Interestingly, Ozkan et al. (24) reported an unusual case of *Babesia bigemina* infection in eight dogs—a pathogen typically associated with cattle.

Etoposide (EP), a topoisomerase II (Top2) inhibitor that induces DNA damage, is clinically employed against small cell lung cancer (25). EP exerts its antitumor effects by preventing DNA re-ligation, thereby inducing critical errors during DNA synthesis in the premitotic phase and promoting cancer cell apoptosis. Its activity is cell cycle-dependent, with predominant effects during the S and G2 phases, mediated specifically through the inhibition of the topoisomerase II alpha isoform (26). However, the clinical utility of etoposide is constrained by its low aqueous solubility, short half-life, and nonspecific toxicities, which collectively limit its bioavailability and therapeutic efficacy. Notably, within the etoposide and cisplatin (EP) regimen, a dose of 500 mg/m^2^ represents a critical threshold beyond which adverse effects—such as alopecia, gastrointestinal toxicity, and acute hypersensitivity reactions—become frequent. For *in vivo* contexts, a previous study reported a murine intraperitoneal LD_50_ of 64 mg/kg (27). The concentration of 500 μM etoposide used in our experiments translates to approximately 125–150 mg/kg *in vivo*, which is considered appropriate for evaluating cytotoxicity in the present study. The limited antiparasitic efficacy of etoposide phosphate (EP) observed in the *B. microti* mouse model, characterized by a non-significant reduction in parasitemia compared to control groups, may be attributed to several factors. Firstly, the dosage used in this study may have been insufficient, as the maximum tolerated dose (MTD) for EP in Balb/C mice was previously established at 75 mg/kg, a regimen associated with distinct weight loss profiles (28). Consequently, higher or optimized dosing regimens might yield stronger antbabesial effects in future investigations. Furthermore, the pharmacokinetic profile of EP likely influenced the weak *in vivo* outcome. Etoposide is known to exhibit low aqueous solubility, a short half-life, and nonspecific toxicities, which collectively limit its bioavailability and efficacy. This is supported by findings from Garg et al. (29), who reported altered etoposide pharmacokinetics in malnourished animals, including a ~ 60% increase in the area under the concentration-time curve (AUC) and decreases in clearance, volume of distribution, and half-life of ~37%, 53%, and 24%, respectively. Additionally, the systemic exposure of etoposide, which is partly metabolized by cytochrome P450 enzyme 3A4 (CYP3A4), can be increased by concomitant administration of CYP3A4 inhibitors such as aprepitant (30).

Protozoan topoisomerases exhibit structural distinctions from mammalian isoforms, presenting potential antiparasitic targets (6). While cellular mechanisms resolving EP-induced Top2-DNA covalent complexes remain incompletely characterized, *Plasmodium falciparum* studies demonstrate EP binding at *Pf*TopoII’s Tyr-829 active site—shared by edotecarin, namitecan, AR-67, and belotecan—inhibiting DNA relaxation by stabilizing the cleavage complex and preventing religation (25, 31). Blood smear analyses suggest EP similarly inhibits religation in *B. bovis*, *B. caballi*, and *T. equi*, inducing irreversible DNA double-strand breaks that trigger apoptosis—consistent with EP’s antitumor mechanism where transient damage becomes permanent (31, 32). Teniposide (VM-26), an EP derivative with analogous activity FDA-approved 32 years ago, remains utilized in pediatric acute lymphoblastic leukemia and gliomas (25).

DNA replication machinery represents a validated target for apicomplexan parasites given its essential role in proliferation (33). Topoisomerase inhibitors are clinically deployed against bacterial infections and malignancies (34), with emerging evidence supporting their utility against Babesia/Theileria species. Ciprofloxacin derivatives, nitidine chloride, and camptothecin exhibit efficacy against these parasites [Tayebwa et al., 2018; (47)], while Plasmodium topoisomerase homologs exist in *Theileria annulata* (34). Notably, apicoplast-localized DNA gyrase (absent in humans) is essential for plastid function; *P. falciparum* GyrA-knockout parasites (ΔPfGyrA) require isopentenyl pyrophosphate (IPP) supplementation and show reduced ciprofloxacin sensitivity but retain EP insensitivity (35). Current babesiosis therapies rely on atovaquone-azithromycin or clindamycin-quinine combinations (36). In this study, EP induced irreversible viability loss in *B. bovis*, *B. caballi*, and *T. equi*—contrasting with transient growth suppression by conventional agents. Species-dependent IC₅₀ variation (highest in *B. bovis*) may reflect differential Top2 expression or structure (3). Morphological alterations including intraerythrocytic spots in *Babesia* spp. and residual structures in *T. equi* align with DNA-damage phenotypes and resemble changes induced by mitochondrial disruptors, hemozoin inhibitors, or dual-mechanism agents like pyronaridine (Top2/hemozoin suppression) and isocoumarins (DNA gyrase/hemozoin inhibition) (37–39). EP (VP-16), as a topoisomerase II inhibitor, exhibits a distinct mechanism of action compared to current anti-Babesial agents such as diminazene (DA), atovaquone combinations (15, 40), or histone deacetylase inhibitors (e.g., panobinostat). While DA demonstrates direct inhibitory effects against *B. bovis* (0.16 ± 0.02 μg/mL), *T. equi* (0.28 ± 0.01 μg/mL), and *B. caballi* (0.012 ± 0.003 μg/mL) (40), EP’s efficacy against apicomplexan parasites remains quantitatively inferior and non-selective. *In vitro* studies indicate that EP’s half-maximal inhibitory concentration (IC₅₀) against *Babesia* spp. exceeds 10 μM (approximately 5.87 μg/mL), which is significantly higher than DA’s potency (0.012–0.28 μg/mL) (15). This contrasts sharply with panobinostat, which shows promising anti-*B. duncani* activity (EC₅₀ 0.0084 μM) and a wide therapeutic window (TC₅₀ 130 μM, SI > 15,000) (41). The primary limitation of EP lies in its mechanism: it stabilizes topoisomerase II-DNA cleavage complexes in rapidly dividing cells, but apicomplexan parasites possess divergent topoisomerase II isoforms with low structural homology to human enzymes.

In terms of safety, the calculated selectivity indices (SI) further support EP’s favorable toxicity profile in mammalian cells. Although EP demonstrated comparatively weaker antiparasitic potency than diminazene aceturate, its higher SI values (ranging from 44 to over 13,000, depending on the species tested) indicate low cytotoxicity to host cells. These results align with the known pharmacological behavior of etoposide, which primarily targets rapidly dividing cells with high Topoisomerase II activity while sparing quiescent cells. Such differential selectivity may allow EP to serve as a scaffold for structure-activity optimization toward parasite-selective analogues. Future work should evaluate EP derivatives or formulation approaches that enhance antiparasitic potency while preserving this advantageous safety profile.

Although *T. annulata* and *B. bovis* encode Topoisomerase IB (6), Top2 remains unconfirmed in Babesia/Theileria, necessitating mechanistic studies on EP-parasite interactions. EP’s irreversible effects prevent recrudescence post-withdrawal, suggesting utility against drug-resistant strains via combination therapies. While nitidine chloride outperformed diminazene aceturate against *B. microti* (47), EP’s lower efficacy here may reflect suboptimal dosing (1 mg/kg vs. 20 mg/kg in prior studies). Prodrug strategies to enhance bioavailability (42) could optimize natural inhibitors like quercetin, which targets protozoan topoisomerases (43). Intriguingly, despite limited parasitological efficacy against *B. microti*, EP significantly improved hematological parameters (RBC, HGB, HCT)—potentially via erythropoietic stimulation during myelosuppression (44) warranting further mechanistic investigation. EP’s differential activity against piroplasms (*B. bovis*, *B. caballi*, *T. equi*) versus *B. microti* may stem from variations in Top2cc repair pathways between protozoan species. Investigating EP’s interaction with parasite DNA repair mechanisms—particularly the presence or absence of homologous recombination components—could reveal novel therapeutic targets. Prodrug strategies (42) may enhance delivery of topoisomerase-targeting compounds like quercetin (43), potentially optimizing EP’s dual role: direct antiparasitic action against susceptible species and hematoprotection in babesiosis.

Although EP showed limited standalone efficacy in this study, its mechanism of targeting topoisomerase II presents potential applicability to other protozoan diseases. Its ability to disrupt the parasite life cycle without regrowth indicates promise in eliminating cryptic parasite stages, a critical factor in preventing chronic or latent infections. Exploring EP’s effectiveness in *Plasmodium* infections, where DNA topoisomerase is a validated target, might provide broader insights into its therapeutic potential across parasitic diseases. Consequently, EP lacks specificity for parasitic targets, leading to off-target toxicity in host cells. Additionally, EP’s immunosuppressive properties could exacerbate Babesiosis-related complications by impairing host defenses, whereas DA or atovaquone-based therapies directly target parasitic metabolic pathways (e.g., mitochondrial electron transport) with higher selectivity. Thus, EP may not a best candidate for Babesiosis treatment, and future efforts should focus on parasite-specific topoisomerase II inhibitors or combination therapies to enhance selectivity and overcome resistance. Therefore, developing inhibitors that specifically target the structural differences between host and parasite topoisomerase II could minimize off-target effects (45). Additionally, leveraging high-throughput screening and structure-based drug design may accelerate the discovery of novel compounds with improved efficacy and safety profiles. Combining such agents with existing antiparasitic drugs might also reduce the likelihood of resistance development, offering a more robust therapeutic strategy against Babesia infections.

The markedly different sensitivities we observed between species (e.g., relatively high IC₅₀ for *B. bovis* versus low IC₅₀ for *B. caballi* and modest *in vivo* activity against *B. microti*) likely reflect multiple, non–mutually exclusive biological and pharmacological factors (46). First, structural and sequence variation in parasite topoisomerase(s) could alter drug binding and stabilization of the TopoII–DNA cleavage complex; even single amino-acid differences at the drug-binding pocket can change affinity and cytotoxic outcome. Second, stage- and species-specific expression levels of TopoII (or alternative topoisomerase isoforms) will modify the fraction of parasites that are vulnerable during S/G₂-like replication phases—species with a higher proportion of actively replicating parasites in culture will appear more sensitive to a cell cycle–dependent poison such as etoposide. Third, differences in DNA damage response and repair capacity (for example, efficiency of homologous recombination, activity of tyrosyl-DNA phosphodiesterases, or other Top2cc resolution pathways) will determine whether TopoII-mediated double-strand breaks are converted into lethal lesions or efficiently repaired. Fourth, variable drug uptake, efflux or intracellular sequestration (including erythrocyte versus parasite membrane permeability, and presence of parasite transporters) and species-specific metabolism could alter intracellular etoposide concentrations. Finally, the *in vivo* pharmacokinetic context (distribution, protein binding, metabolism by host CYPs, and intrared blood cell bioavailability) differs from *in vitro* exposure and can reduce effective concentration at the parasite. Collectively, these mechanisms provide plausible explanations for why etoposide produced irreversible *in vitro* effects in some piroplasms yet only modest *in vivo* efficacy against *B. microti*. Future mechanistic studies—including comparative TopoII sequencing and structural modelling, recombinant enzyme inhibition assays, expression profiling of topoisomerases and DNA-repair genes, and measurements of intra-erythrocytic drug accumulation-will be required to pinpoint the dominant determinants of species-specific sensitivity and to inform rational design of parasite-selective TopoII inhibitors.

## Data Availability

The original contributions presented in the study are included in the article/Supplementary material, further inquiries can be directed to the corresponding authors.
