# Supplementary material for: Leveraging topoisomerase II-mediated DNA damage: repurposing etoposide as a lead compound for apicomplexan parasite control
Source: Front Vet Sci. 2025 Nov 19;12:1689833. doi: 10.3389/fvets.2025.1689833 (PMC12673203; doi:10.3389/fvets.2025.1689833)
Supplement: Supplementary file 1 [file Presentation_1.pptx]

## Slide 1
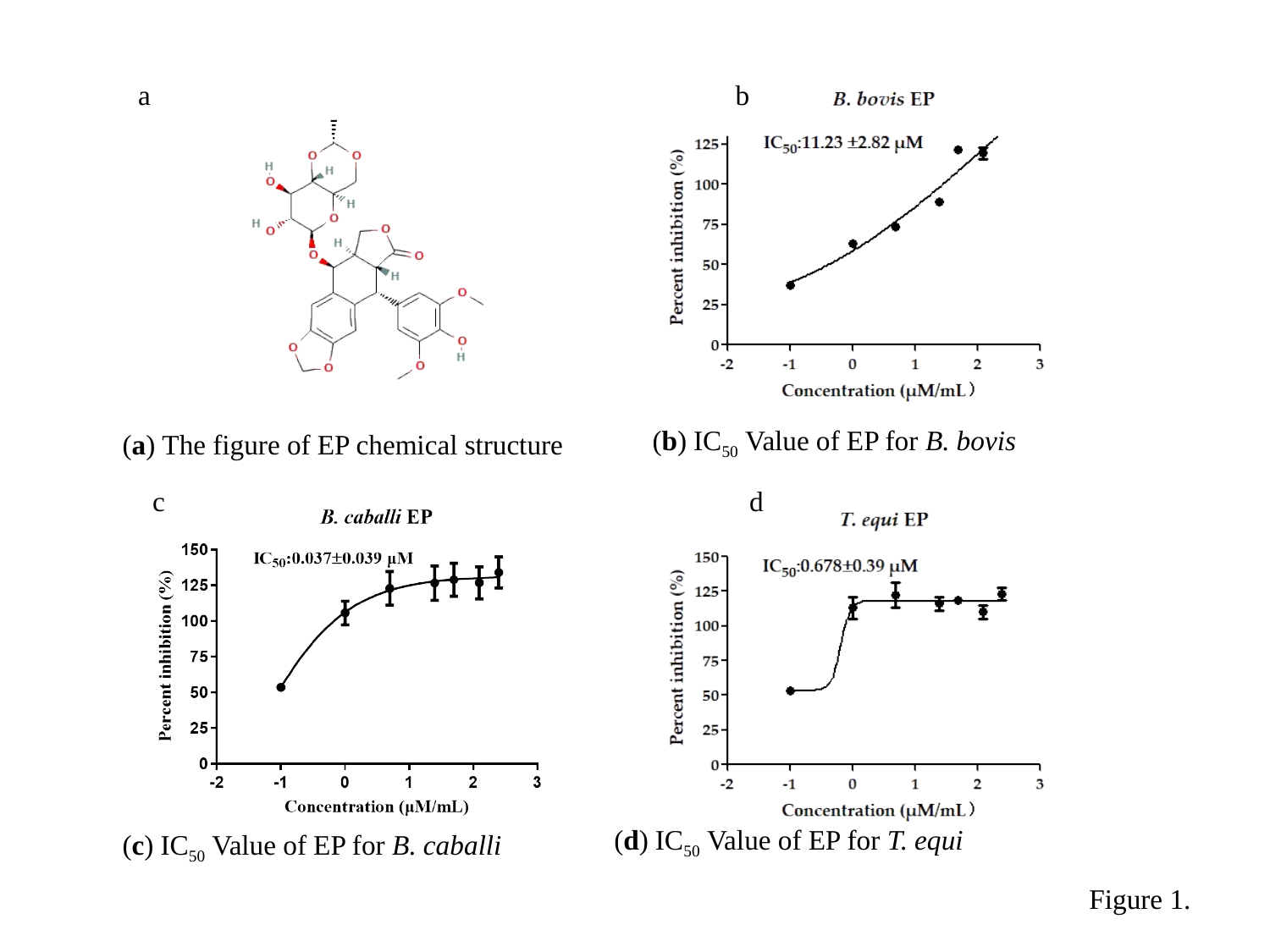

a
b
c
d
(a) The figure of EP chemical structure
(b) IC50 Value of EP for B. bovis
(d) IC50 Value of EP for T. equi
(c) IC50 Value of EP for B. caballi
Figure 1.

## Slide 2
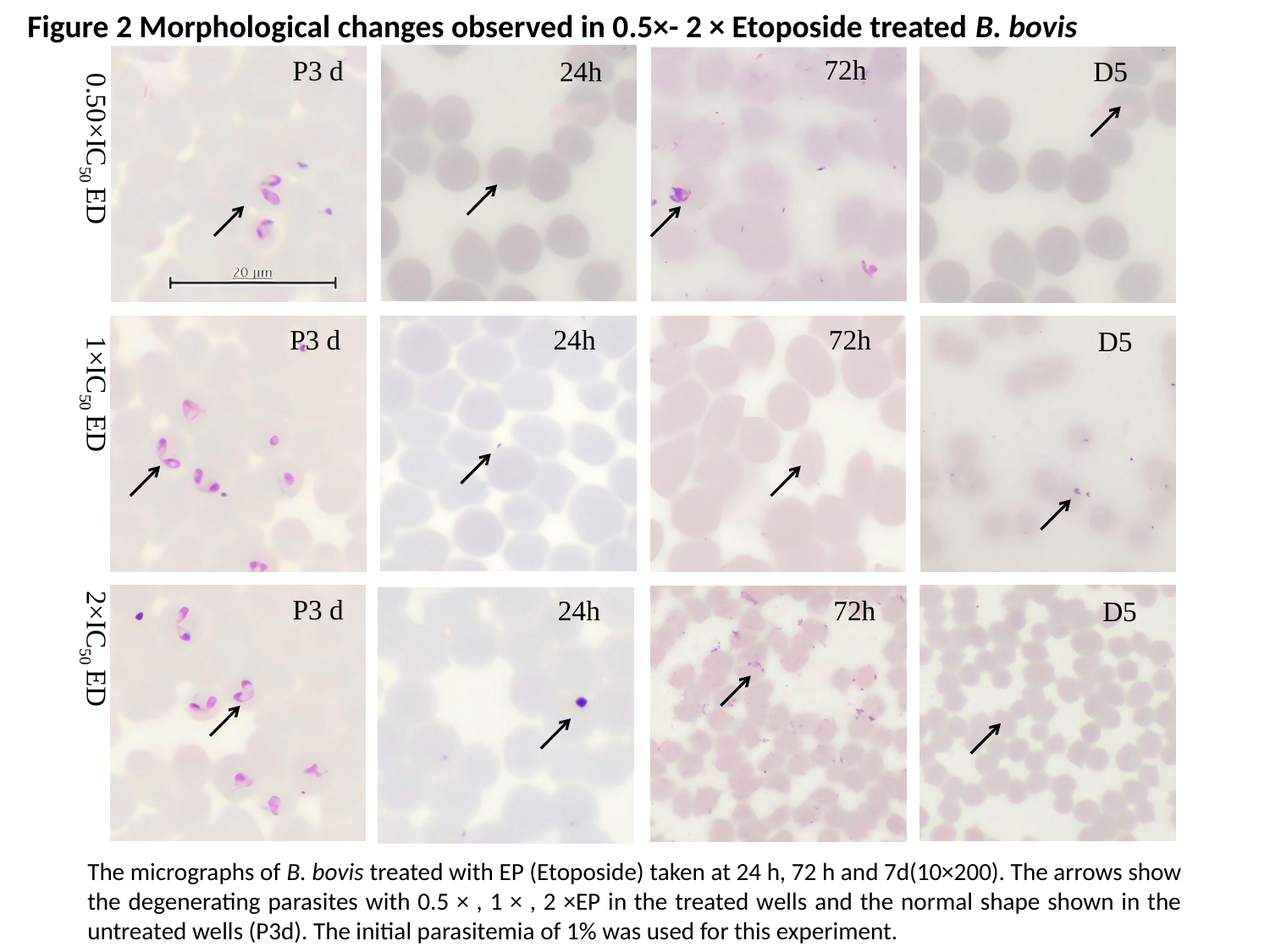

Figure 2 Morphological changes observed in 0.5×- 2 × Etoposide treated B. bovis
72h
P3 d
P3 d
P3 d
24h
D5
0.50×IC50 ED
24h
72h
D5
1×IC50 ED
2×IC50 ED
24h
72h
D5
The micrographs of B. bovis treated with EP (Etoposide) taken at 24 h, 72 h and 7d(10×200). The arrows show the degenerating parasites with 0.5 × , 1 × , 2 ×EP in the treated wells and the normal shape shown in the untreated wells (P3d). The initial parasitemia of 1% was used for this experiment.

## Slide 3
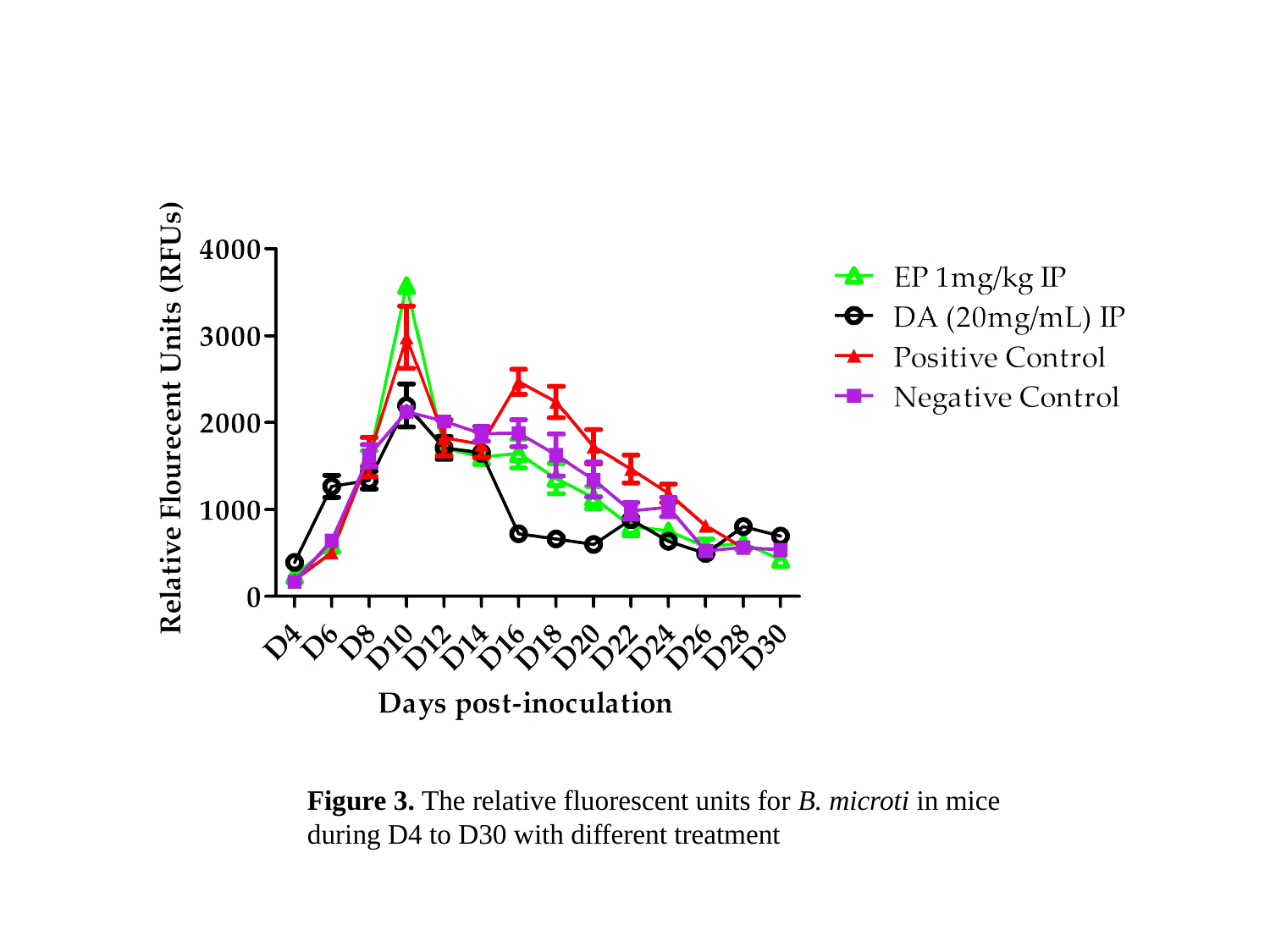

Figure 3. The relative fluorescent units for B. microti in mice during D4 to D30 with different treatment

## Slide 4
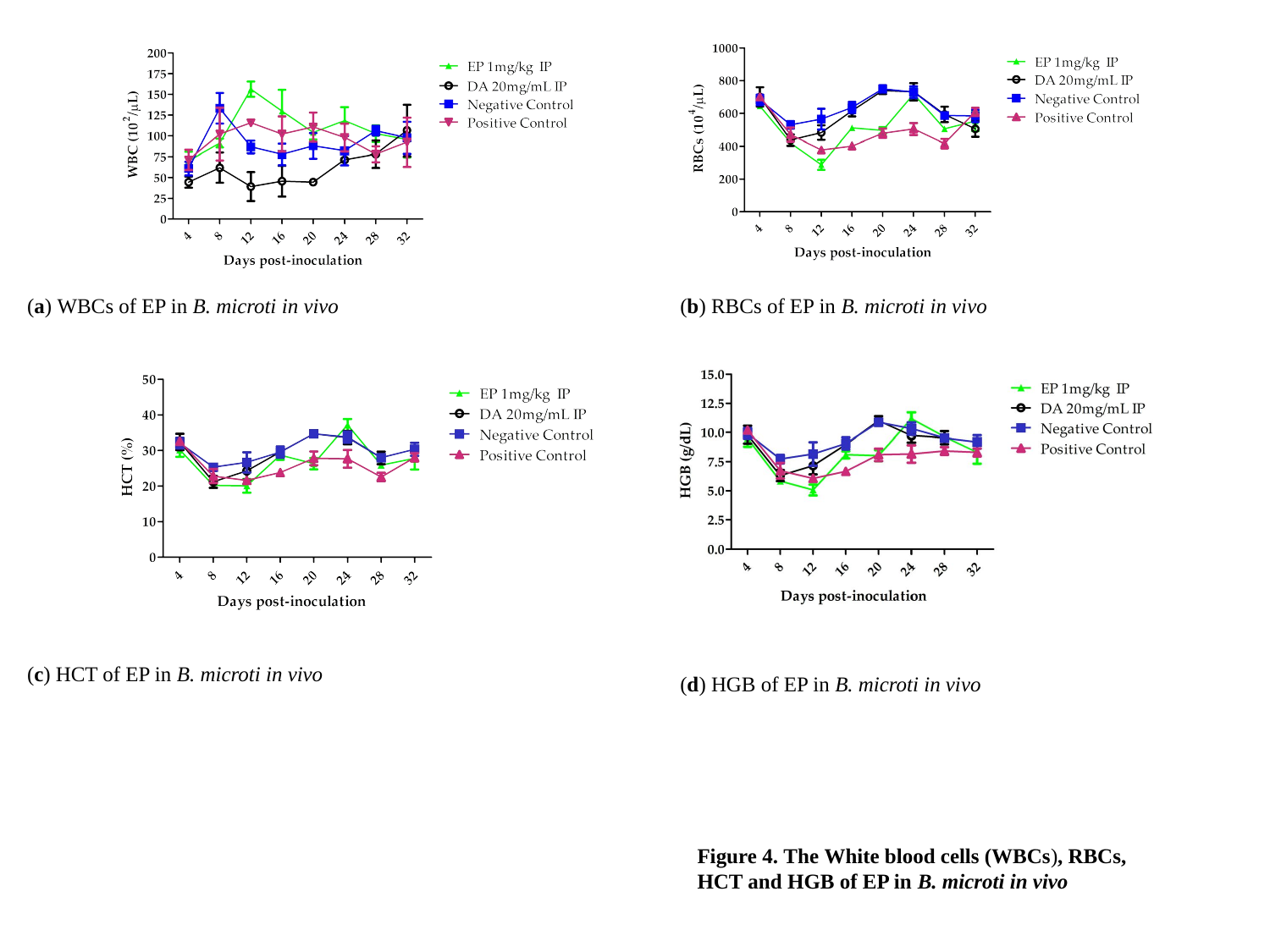

(b) RBCs of EP in B. microti in vivo
(a) WBCs of EP in B. microti in vivo
(c) HCT of EP in B. microti in vivo
(d) HGB of EP in B. microti in vivo
Figure 4. The White blood cells (WBCs), RBCs, HCT and HGB of EP in B. microti in vivo
